# Conformity in numbers—Does criticality in social responses exist?

**DOI:** 10.1371/journal.pone.0209620

**Published:** 2018-12-27

**Authors:** Piotr Nyczka, Katarzyna Byrka, Paul R. Nail, Katarzyna Sznajd-Weron

**Affiliations:** 1 Department of Life Sciences & Chemistry, Jacobs University Bremem, Bremem, Germany; 2 Faculty of Psychology in Wrocław, University of Social Sciences and Humanities, Wrocław, Poland; 3 Department of Psychology and Counseling, University of Central Arkansas, Conway, United States of America; 4 Faculty of Fundamental Problems of Technology, Wrocław University of Science and Technology, Wrocław, Poland; Coventry University, UNITED KINGDOM

## Abstract

Within this paper we explore the idea of a critical value representing the proportion of majority members within a group that affects dramatic changes in influence targets’ conformity. We consider the threshold *q*-voter model when the responses of the Willis-Nail model, a well-established two-dimensional model of social response, are used as a foundation. Specifically, we study a generalized threshold *q*-voter model when all basic types of social response described by Willis-Nail model are considered, i.e. conformity, anticonformity, independence, and uniformity/congruence. These responses occur in our model with complementary probabilities. We introduce independently two thresholds: one needed for conformity, as well as a second one for anticonformity. In the case of conformity, at least *r* individuals among *q* neighbors have to share the same opinion in order to persuade a voter to follow majority’s opinion, whereas in the case of anticonformity, at least *w* individuals among *q* neighbors have to share the same opinion in order to influence voters to take an opinion that goes against that of their own reference group. We solve the model on a complete graph and show that the threshold for conformity significantly influences the results. For example, there is a critical threshold for conformity above which the system behaves as in the case of unanimity, i.e. displays continuous and discontinuous phase transitions. On the other hand, the threshold for anticonformity is almost irrelevant. We discuss our results from the perspective of theories of social psychology, as well as the philosophy of agent-based modeling.

## Introduction

In 2008 Nolan et al. reported a series of very interesting social experiments on energy conservation [1]. In one of their studies they examined different potential influence sources related to either self-interest (viz., saving money) or social responsibility (viz., protecting the environment) in hopes of changing individual’s behavior regarding using less electricity. However, neither of these sources worked as well as a simple message/ a descriptive norm saying: “In a recent survey of households in your community, researchers at Cal State San Marcos found that 77% of San Marcos residents often use fans instead of air conditioning to keep cool in the summer. Using fans instead of air conditioning—Your Community’s Popular Choice!” [1]. These findings indicate that what the majority is doing, or at least what one thinks the majority is doing, can be more influential in changing behavior than inherent rewards from either financial incentives or from safeguarding the environment.

In the present article, however, our focus is not on the power of social forces in influence, which has already been abundantly established, but rather the size of the majority relative to the minority. In the Nolan et al. research, the majority percentage was 77%. Similar values (75%) appeared in field experiments on descriptive norms designed to increase hotel guests’ towel reuse [2, 3]. However, it has been argued that a descriptive norm of, say, 90% to German hotel clients would be more appropriate. We would like to stress here that the above percentages are not real majorities that save energy or reuse towels. Rather, these are values chosen by researchers merely as independent variables for the purposes of research. This point is probably entirely clear for sociologists or social psychologists, but it may not be clear for physicists, mathematicians, etc.

In the present research, we manipulated the majority percentage within the group of influence across a very wide range of values to examine its effects; yet, to simplify our presentation, we focus on the values of 50%, 75%, and 100%.

Intuitively, larger majorities should be more influential than smaller ones because they bring to bear more social pressure on the targets of influence, more weight, so to speak. However, is there a critical value that yields dramatic changes in influence, below which the majority has little impact and above which an increase in the majority’s size yield negligible additional influence? An example from physical science may help to clarify the idea of a critical value.

It is common knowledge that under the normal pressure water becomes ice at 0 degrees Celsius and vapor at 100 degrees. So, between 0 and 100 it is just water, colder or warmer, but still just water. Then suddenly at 0 it starts to freeze and at 100 begins to boil. Zero and 100 degrees are the points of so called discontinuous phase transitions, at which probabilities of both phases (e.g. solid and liquid) are equal. It is possible to supercool water so that it stays liquid even in negative temperatures. However, a critical temperature exists, below which the water absolutely must freeze. There are many additional examples of such critical points, for example, traffic jams occur only above a certain critical density of cars on the road. So now we ask the question: Is there any particular percentage in terms of the size of the majority above which a social influence system starts to behave dramatically differently in terms of social impact and conformity?

Social psychologists have long searched for critical values or “magic numbers” that could capture the effects of the group on the individual [4]. For example, Slater found that the optimum group size, specifically the most effective number of people in a group when dealing with an intellectual task, is about five [5]. Furthermore, Asch found that a majority/minority size of three versus one is sufficient for the full social impact of the majority to be felt by a lone target when under implicit pressure to conform. However, Asch also showed that for this effect to occur it is essential that the majority be unanimous [6, 7]. With a majority group size of six, a single dissenter reduced the overall conformity rate for a lone target from about 33% to 5%.

Latané argued, on the other hand, that the larger the group up to a certain point, the greater its impact. Specifically, the function relating group size to conformity is a negatively accelerating growth curve [8]. A related idea proposed by Mullen explains the effect of group size on conformity in terms of self-attention theory [4]. Mullen defined the Other-Total Ratio (OTR) as the number of people in the other subgroup (i.e. outside of the individual’s own group) relative to the number of people in a total group. He has shown that this ratio accurately predicts group members’ levels of self-attention, as well as their levels of conformity [4, 9]. Specifically, as OTR increases, conformity increases as well.

Considering all of this evidence, we wonder with respect to OTR “Is there any *magic number* that maximizes the degree of a group’s influence or causes some qualitative change on the macroscopic level (i.e. observable in the society)?” Nolan et al. used a majority of size 77% to motivate people to use less energy. We propose that this 77% can be described from the perspective of self-attention theory using the idea of OTR. If we assume that a person receiving this information accepts that 77% of neighbors (the other subgroup) use less energy, while he/she is among the 23% of people that use more energy (one’s own subgroup), then *OTR* = 0.77. Two questions could be asked: Is OTR = 0.77 somehow critical? If so, in what sense?

It seems desirable to determine, therefore, whether a critical value of a majority or a critical value of *OTR* exists. Furthermore, if it exists, is it universal or does it perhaps depend upon the size of the influence group, the type of the social influence, etc.? Determining the answer to such questions on the basis of empirical research would be the most appropriate and convincing strategy. Yet, it would also be very demanding and beyond our and most researchers’ present capabilities. However, we can at least examine whether such a critical value could potentially exist using the theoretical, agent-based model (ABM). Such a strategy is the focus of the present paper. We will introduce ABM based on one of the most precise and complete yet simple models of social response, namely the Willis-Nail two-dimensional model. We demonstrate at the macroscopic level that it leads to unexpected, yet psychologically interpretable results, ones that potentially could be tested in social experiments, a task for the future. We also hope that our results will convince those who doubt the validity of ABM by showing that social simulations go beyond “you get out what you put in” [10].

## 1 The basis of the model

Within this paper we consider and integrate the threshold *q*-voter model (qVM) [11, 12] with the Willis-Nail two-dimensional model of social response [13–17]. The agent-based model (ABM) we propose here takes its roots in the Sznajd model [18, 19], in which the idea of unanimity for conformity has been introduced. The Sznajd model, next to the voter model [20, 21], the social impact model [22, 23], majority model [24, 25] and the threshold model [26], is one of the ABM, in which agents are described by the two-state dynamical variables (dichotomous opinions: YES/NO, AGREE/DISAGREE etc.) and interactions between agents are based on conformity. Within the Sznajd model, only a unanimous group of agents can persuade neighbors to take the opinion shared by the group of influence. The size of the group depends of the graph’s structure, for example on a one dimensional ring it consists of two agents. However, already for the two-dimensional square lattice, several versions have been proposed, e.g. in the so-called Stauffer generalization, the group consists of four agents [19], but in Galam’s and Kondrat’s generalizations it consists always of two agents, no matter the structure of a graph [27].

The idea of unanimity for conformity has been later adopted under the more general framework of the *q*-voter model [28]. Unlike the Sznajd model, the original *q*-voter model is clearly defined on an arbitrary graph and *q* is the parameter of the model, which is independent of the graph’s structure. Within this model the system consists of *N* agents (voters), which are placed in the nodes of a certain graph of size *N*—each node of a graph is occupied by exactly one agent. Agents are described by binary dynamical variables *S*_*i*_(*t*) (usually called opinions), where *i* = 1, …, *N* labels nodes and *t* is time measured usually in Monte Carlo Steps, which means elementary time step Δ*t* = 1/*N*. At each time *t* one agent, placed at site *i*, is chosen randomly from the whole system. Next *q* neighbors, i.e. nodes which are directly linked with the site *i*, are chosen also randomly. Of course, it may happen that node *i* is linked with less than *q* sites and thus originally neighbors were chosen repetitively. If all *q* opinions are the same, then a voter at site *i* also takes the same opinion as chosen neighbors, i.e. *S*_*i*_(*t* + Δ*t*) = *S*_*j*_(*t*), where *j* labels one of the *q* agents. Otherwise it flips to the opposite state, i.e. *S*_*i*_(*t*+ Δ*t*) = −*S*_*i*_(*t*), with probability *ϵ*. For *ϵ* = 0 we obtain a rule “if you do not know what to do, just do nothing”, introduced for the Sznajd model [18] and such a rule, i.e. *ϵ* = 0, was used in most of later publications on the *q*-voter model [11, 29–40].

**Fig 1:**
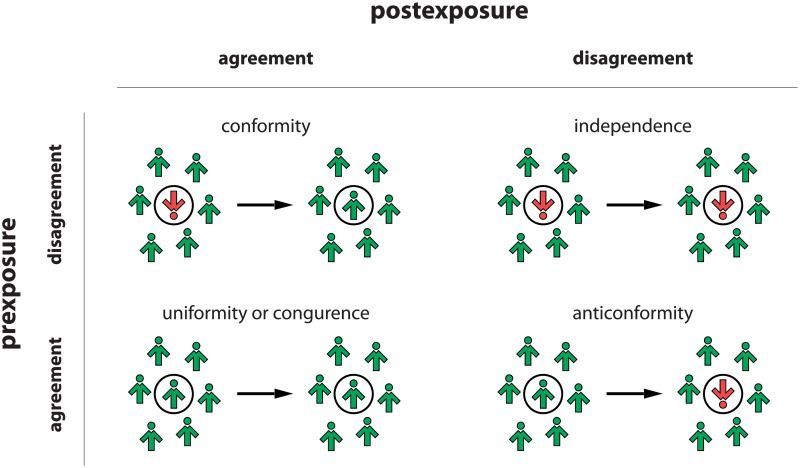
The four possible responses to social influence of the Willis-Nail two-dimensional model.

In the original *q*-voter model, only one type of social response, that has potential to change the state of the system, was introduced, namely conformity. Another type of social response, i.e. uniformity/congruence, is present in all ABMs of opinion dynamics, but it does not change the state of the system, as shown in Fig 1. This type of response means that one does not change one’s opinion because before, as well as after exposure to the group, the individual has the same opinion as the group. Later on, two other types of social response were incorporated into the model, namely independence [11, 30, 33, 35, 36, 38, 39] and anticonformity [11, 30, 34, 37, 40].

In this paper, we will follow the path that starts in [11, 30], which means that agents will be homogeneous (a so called situation approach [41, 42]), as opposed to research by Mobilia etal. [33, 36, 38]. Moreover, as in [11, 30, 41, 42] we assume that in the case of independence the agent can randomly change her/his opinion to the opposite one, independently of the neighborhood, with probability *f*, which is the same idea as in the noisy voter model [43–46]. On the contrary, Mobilia introduced to the *q*-voter so called zealots, i.e. agents that never change their opinion [33, 36, 38, 47], which corresponds to *f* = 0. Herein we set *f* = 1/2, because it has been shown that *f* > 0 only rescales results [48]. The novelty of the approach proposed in this paper is that in contrast to all earlier papers [11, 30, 35, 39, 41, 42], we examine the model with all basic types of social response as described by the Willis-Nail two-dimensional model. This means that our model will consider simultaneously four types of response, i.e. conformity, anticonformity, independence, and uniformity/congruence. As such, it will reduce previously considered models to special cases of the present model.

## 2 The model

As in the original *q*-voter model [28] and its later modifications [11, 30, 33, 34, 36, 38], we consider *N* agents placed in the nodes of an arbitrary graph. Each node of a graph is occupied by exactly one agent. If two nodes are linked then agents in these nodes can influence each other. Here we will focus on unweighted and undirected graphs. Moreover, we will consider only fully-connected graphs (complete graphs). The chosen topology implies that all individuals can interact with each other. Although such structures are too simple to model large social networks, they are decent representations of interactions within small groups and will allow for us an analytical treatment [11, 28, 30, 33]. In the future, it would be also interesting to consider more complex graphs. In this context, a recent idea of a modified version of the Erdös-Renyi (ER) graph (so called ER-L, from Erdös-Renyi-like), devised for dilution processes, seems to be promising [49]. Within the ER-L model, depending on model’s parameters, the graph can split into two communities or remain connected, which makes it very interesting from a social-psychological point of view.

**Fig 2:**
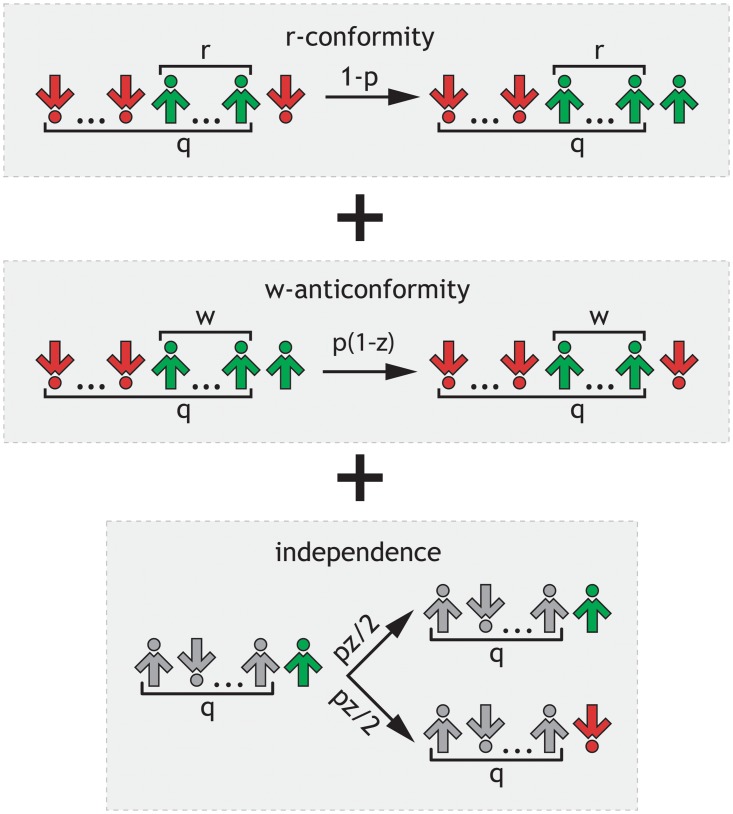
Schematic illustration of the threshold *q*-voter model with two types of nonconformity. In the case of independence the reference group is denoted by a gray color to stress an absence of influence. A voter does not change under peer pressure but takes one of two positions randomly with the same probability *f* = 1/2.

As in all versions of the *q*-voter model, each agent is described by a dynamical binary variable *S*_*i*_(*t*) = ±1, *i* = 1, 2,…, *N*. However, in this work we consider simultaneously all four possible responses to social influence of the Willis-Nail two-dimensional model, which was derived from Willis’s symbolic scheme [13] and formalized by Nail et al. [15, 16, 50] (see Fig 1). This means that at each elementary time step we choose randomly a target individual and a type of social response that will be provided by this individual in a given time step: nonconformity takes place with the probability *p*, whereas conformity with the complementary probability 1 − *p*. Moreover, in the case of nonconformity, there are two specific sub-types: (a) independence with probability *z* and (b) anticonformity with complementary probability 1 − *z*. In summary, one of the three reposes is possible: (1) conformity with probability 1 − *p*, (2) anticonformity with probability *p*(1 − *z*) or (3) independence with probability *pz* (see Fig 2).

In case of conformity and anticonformity, a chosen target will be influenced by a source of *q* individuals randomly chosen from the whole system. As already mentioned, in the original model and its later modifications, only a unanimous group could influence a target, yet more recently a generalized model has been introduced in which a threshold *r* was needed to influence a target [11, 12]. Here we will use this generalized version, which means that conformity takes place if at least *r* agents among *q* are in the same state. Furthermore, anticonformity takes place only if at least *w* agents among *q* are in the same state and in general *w* ≠ *r*.

On the one hand, such a generalized model is unfortunately described by up to five parameters: (1) the size of a source of influence *q*, (2) the probability of nonconformity *p*, (3) the conditional probability that in case of nonconformity a target behaves independently *z*, (4) the threshold needed for conformity *r*, (5) the threshold needed for anticonformity *w*. On the other hand, such a model contains as special cases all earlier models considered by Nyczka et al. [11, 30]. Not going further into details, let us recall here that two models with *z* = 1, *r* = *w* = *q* and *z* = 0, *r* = *w* = *q* were considered [30]. Later on, four models with *z* = 1, *r* = *w* > *q*/2, *z* = 0, *r* = *w* > *q*/2, *z* = 1, *r* > *q*/2, *w* = *q* and *z* = 0, *r* > *q*/2, *w* = *q* were considered in [11]. However, so far the model with *z* ∈ (0, 1), i.e. with both types of nonconformity (independence and anticonformity) has not been studied. Yet, only such a model can be treated as a realistic model of social response, this given the actuality of conformity, independence, anticonformity and uniformity/congruence in real-world influence settings.

## 3 Methods

Due to the social interactions, the system evolves over time and finally reaches the steady state. In the *q*-voter model without nonconformity, the system eventually always reaches consensus, i.e. all agents have the same opinion [31, 32]. This is not surprising, because in this case the only type of social response is conformity. In the *q*-voter model with conformity and only one type of nonconformity, i.e. for either *z* = 1 (conformity + independence) or *z* = 0 (conformity + anticonformity), two types of steady states are possible depending on the model’s parameters. This means that, as a result of competition between conformity and nonconformity, an order-disorder phase transition is observed for a critical value of *p* = p*, which depends on *q* and the type of nonconformity. To quantify this phase transition, as is usually true in these types of models, an average opinion (magnetization, hence symbol *m*, in the language of spin models) is introduced as an order parameter [30]:

$$\begin{array}{c} m=\frac{N_{↑}-N_{↓}}{N}=\frac{1}{N}\sum_{i=1}^{N}S_{i}, \end{array}$$ (1)

where *N* is the number of agents in the system, *N*_↑_ is the number of agents with positive opinion and *N*_↓_ is the complementary number of agents with negative opinion.

It has been shown that a the low level of nonconformity *p* < p* the majority of people have the same opinion, i.e. *m* ≠ 0, which means that in case of voting the society will point clearly to one of two possible choices. On the other hand, for a high level of nonconformity *p* > p* there will be a stalemate situation, i.e. average opinion *m* will oscillate around zero, so in case of voting the results will be in a sense accidental. Interestingly, the dependence between p* and *q* is completely different for each type of nonconformity: for independence p* decreases with *q* but for anticonformity p* increases with *q*. Moreover, for independence the transition is continuous for 2 ≤ *q* ≤ 5 but discontinuous for *q* ≥ 6, whereas for anticonformity it is continuous in both cases [11, 30].

Because we consider the model on the complete graph and agents are homogeneous, we can completely describe the state of the system by using only the average opinion *m*. We consider so-called random sequential updating schemes, which means that in every elementary time step, only a single agent can have a change of opinion. Therefore, there are only three possible scenarios: the total number of agents with a positive opinion *N*_↑_ may increase by 1, decrease by 1, or remain unchanged. This corresponds to a change in the public opinion *m* by 2/*N*. Each of these scenarios occurs with another transition rate that can be easily calculated analytically, provided we consider the model on a complete graph.

Having transition rates, we can write down the rate equation, which allows us not only to calculate the stationary value of average opinion *m*, but also to account for its evolution over time [30]. Moreover, we can also write down the Master equation, which allows us to calculate the probability density function of *m*, thereby helping to distinguish between different types of phase transitions. Alternatively, having transition rates we can introduce an effective potential and then apply the Landau approach [51], what has been done in case of the *q*-voter model with one type of nonconformity [30]. This latter method is very simple and effective. Moreover, it will allow us not only to calculate critical values of parameters but also to determine the type of phase transition.

For the *q*-voter model with two types of nonconformity, the calculations are, of course, more tedious than for models with only one type of nonconformity, and the final formulas longer. Nevertheless, they can still be presented in a relatively compact way, and therefore we decided to present them here. We have moved to the S1 Appendix only the part connected to Landau’s approach.

In the generalized model, the opinion can change due to one of three types of social response. Let us denote by *α*^±^, *β*^±^ and *γ*^±^ the conditional probabilities of conformity, anticonformity, and independence respectively in the case of choosing a particular type of social interaction. As mentioned previously, uniformity/congruence does not change the state of the system, and thus, we do not need to introduce any notation for the probability of this response. Nevertheless, it can be easily calculated if needed because the probabilities of different social responses are complementary, i.e. add up to 1. Indexes + and − are respectively for increasing or decreasing an average opinion *m*. For example, *α*^−^ is the conditional probability of decreasing of *m* due to conformity and *γ*^+^ is the conditional probability of increasing *m* due to independence. Within this notation, we can write down the transition rates for all three possible events that can occur in an elementary time step Δ*t*:

$$\begin{array}{c} m\left(t+\Delta t\right)=\{\begin{array}{ccc} m\left(t\right)-\frac{2}{N} & \text{with} & \lambda^{-},\\ m(t) & \text{with} & \lambda^{0},\\ m\left(t\right)+\frac{2}{N} & \text{with} & \lambda^{+}, \end{array} \end{array}$$ (2)

where

$$\begin{array}{ccc} \lambda^{+} & = & (1-p)\cdot \alpha^{+}+p\left(1-z\right)\beta^{+}+pz\cdot \gamma^{+},\\ \lambda^{-} & = & (1-p)\cdot \alpha^{-}+p\left(1-z\right)\beta^{-}+pz\cdot \gamma^{-},\\ \lambda^{0} & = & 1-\lambda^{+}-\lambda^{-}, \end{array}$$ (3)

where probabilities of opinion growth *α*^+^ and decay *α*^−^, related to conformity are given by:

$$\begin{array}{ccc} \alpha^{+} & = & \sum_{i=r}^{q}\left(\begin{array}{c} q\\ i \end{array}\right)\frac{N_{↓}\prod_{j=1}^{i}\left(N_{↑}-j+1\right)\cdot \;\prod_{j=1}^{q-i}\left(N_{↓}-j\right)}{\prod_{j=1}^{q+1}\left(N-j+1\right)},\\ \alpha^{-} & = & \sum_{i=r}^{q}\left(\begin{array}{c} q\\ i \end{array}\right)\frac{N_{↑}\prod_{j=1}^{i}\left(N_{↓}-j+1\right)\;\cdot \prod_{j=1}^{q-i}\left(N_{↑}-j\right)}{\prod_{j=1}^{q+1}\left(N-j+1\right)}, \end{array}$$ (4)

probabilities of opinion growth *β*^+^ and decay *β*^−^, related to anticonformity are given by:

$$\begin{array}{ccc} \beta^{+} & = & \sum_{i=r}^{q}\left(\begin{array}{c} q\\ i \end{array}\right)\frac{N_{↓}\prod_{j=1}^{i}\left(N_{↓}-j\right)\cdot \prod_{j=1}^{q-i}\left(N_{↑}-j+1\right)}{\prod_{j=1}^{q+i}\left(N-j+1\right)},\\ \beta^{-} & = & \sum_{i=w}^{q}\left(\begin{array}{c} q\\ i \end{array}\right)\frac{N_{↑}\prod_{j=1}^{i}\left(N_{↑}-j\right)\cdot \prod_{j=1}^{q-i}\left(N_{↑}-j+1\right)}{\prod_{j=1}^{q+i}\left(N-j+1\right)} \end{array}$$ (5)

and probabilities of opinion growth *γ*^+^ and decay *γ*^−^, related to independence are equal:

$$\begin{array}{ccc} \gamma^{+} & = & \frac{N_{↓}}{2N},\\ \gamma^{-} & = & \frac{N_{↑}}{2N}. \end{array}$$ (6)

For the infinite system formulas for *α*^±^, *β*^±^ and *γ*^±^ are much simpler, i.e.

**conformity**:
  $$\begin{array}{ccc} \alpha^{+} & = & \sum_{i=r}^{q}\left(\begin{array}{c} q\\ i \end{array}\right){\left(\frac{1+m}{2}\right)}^{i}{\left(\frac{1-m}{2}\right)}^{q-i+1},\\ \alpha^{-} & = & \sum_{i=r}^{q}\left(\begin{array}{c} q\\ i \end{array}\right){\left(\frac{1-m}{2}\right)}^{i}{\left(\frac{1+m}{2}\right)}^{q-i+1}. \end{array}$$ (7)
**anticonformity**:
  $$\begin{array}{ccc} \beta^{+} & = & \sum_{i=w}^{q}\left(\begin{array}{c} q\\ i \end{array}\right){\left(\frac{1-m}{2}\right)}^{i+1}{\left(\frac{1+m}{2}\right)}^{q-i},\\ \beta^{-} & = & \sum_{i=w}^{q}\left(\begin{array}{c} q\\ i \end{array}\right){\left(\frac{1+m}{2}\right)}^{i+1}{\left(\frac{1-m}{2}\right)}^{q-i}. \end{array}$$ (8)
**independence**:
  $$\begin{array}{ccc} \gamma^{+} & = & \frac{1-m}{4},\\ \gamma^{-} & = & \frac{1+m}{4}. \end{array}$$ (9)

Having all the above formulas we could calculate the time evolution of average opinion and even the evolution of probability density function of *m*, but here we are only interested in the dependence between the average opinion and the model’s parameters in the stationary state. Such a relation can be easily obtained from the condition of stationarity, which means that the rate of opinion growth should be equal to the rate of opinion decay:

$$\begin{array}{c} \lambda^{+}=\lambda^{-}. \end{array}$$ (10)

Thus from Eq (3) we obtain:

$$\begin{array}{c} (1-p)\cdot \alpha^{+}+p(1-z)\cdot \beta^{+}+pz\cdot \gamma^{+}=(1-p)\cdot \alpha^{-}+p(1-z)\cdot \beta^{-}+pz\cdot \gamma^{-}. \end{array}$$ (11)

We cannot calculate analytically the function *m* = *m*(*p*) from the above formula, but we can easily derive the opposite relation *p* = *p*(*m*) and then rotate the plot. Such a procedure has been done already for the *q*-voter model with one type of nonconformity [30]. Furthermore, dependence *p* = *p*(*m*) allows us to calculate the critical value of *p* = p* and determine the type of the phase transition. As we will see in the next section, function *p* = *p*(*m*) in the stationary state will have always an extreme at *m* = 0: minimum for continuous phase transitions and maximum for discontinuous phase transitions. Thus, to determine the type of the phase transition, we calculate the second derivative:

$$\begin{array}{c} {\left[\frac{\partial^{2}p}{\partial m^{2}}\right]}_{m=0}\{\begin{array}{cc} <0 & \text{continuous}\;\text{transition}\\ >0 & \text{discontinuous}\;\text{transition} \end{array} \end{array}$$ (12)

Unfortunately, in our case at *m* = 0 there is a singularity and even the L’Hospital’s rule don’t help much here. Therefore, to determine the critical points and the type of the phase transition we have used Landau’s approach [51], like in [30]. To use this approach we need an effective potential, defined as:

$$\begin{array}{ccc} V(m) & = & -\int F(m)dm, \end{array}$$ (13)

where

$$\begin{array}{ccc} F & = & \lambda^{+}-\lambda^{-}. \end{array}$$ (14)

can be treated as an effective force, in fact a drift as shown in [52]: λ^+^ drives the system to the state with positive opinions, whereas λ^+^ to the one with negative opinions. Of course, an effective force and all probabilities, including λ^±^, *α*^±^, *β*^±^, *γ*^±^ are functions of the order parameter *m*, which results from the Eqs (3) and (7)–(9), but we did not write it explicitly to keep the notation short. Because exact calculations using Landau’s approach are rather technical, and will be probably interesting only for some readers, we have moved them to S1 Appendix.

## 4 Results

The question we asked in the Introduction was if any critical value of other-total ratio related to conformity exists. In our model there are two parameters related to the other-total ratio needed for social influence: (1) *r*, i.e. the number of individuals with the same opinion inside of the group of influence needed for conformity and (2) *w*, i.e. the number of individuals with the same opinion inside of the group of influence needed for aniconformity. Thus, within our ABM the question about the critical majority needed for conformity is the question about the critical value of *r*.

**Fig 3:**
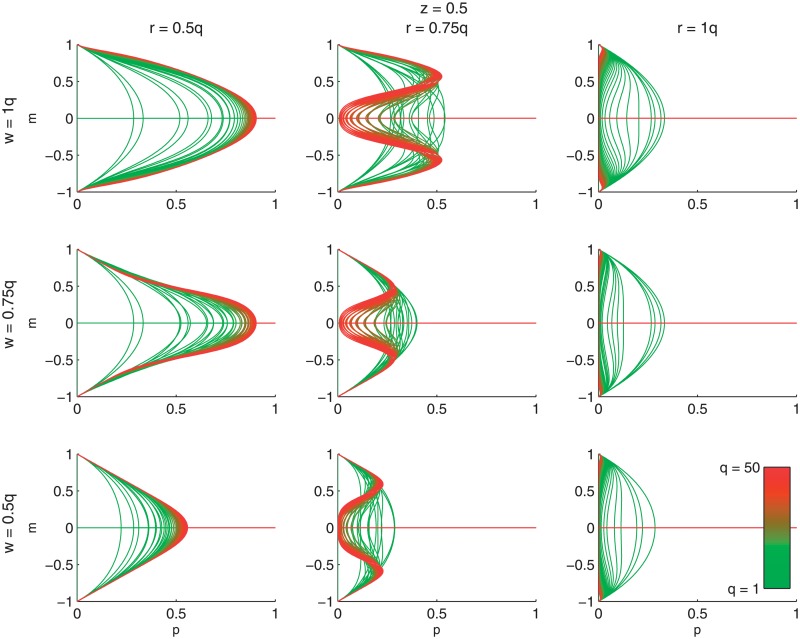
Dependence between the public opinion *m* and the probability of nonconformity *p* within the threshold *q*-voter model with two types of nonconformity.

Let us start by presenting the dependence between an average opinion *m* and the level of nonconformity *p* for different values of *r*, *w* and the size of influence group *q*. Noteworthy, we have one more parameter in our model, which describes the conditional probability of being independent in the case of nonconformity, namely *z*. So far only *z* = 0 and *z* = 1 have been studied [11, 30], so here we start with *z* = 1/2, which means that both types of nonconformity are equally probable. We see in Fig 3 that also in our generalized model, we have a phase transition analogously as in earlier versions of the *q*-voter model. For small values of *p* < p* average opinion *m* ≠ 0 and for *p* > p* average opinion *m* = 0. It is seen that critical value p* depends on model’s parameters *r*, *w* and *q*.

Strikingly, the parameter *r*, related to conformity, influences the results much more strongly than the parameter *w*, related to anticonformity. It is seen when we compare the difference between subplots in Fig 3: subplots within each column (i.e. for the same value of *r* but different values of *w*) are qualitatively similar to each other, on the other hand, they differ within each row. For example, for *r* = 0.5*q* (first column) function *p*(*m*) has a single minimum at *m* = 0, which indicates continuous phase transition. For *r* = 0.75*q* we see already two types of behavior: for smaller values of *q* < q* the function *p*(*m*) has a single minimum at *m* = 0, whereas for larger values of *q* > q* there are two minima and one maximum at *m* = 0, which indicates discontinuous phase transition. In the last column (*r* = *q*) the situation is similar, i.e. both types of phase transitions are seen, but everything shifts towards zero and thus is not easily seen in this scale. We decided to keep the same scale in all subplots to visualize more clearly the similarities between plots within each column and differences in each row.

Summarizing, Fig 3 indicates that as long as the value of *r* (majority needed for conformity) is fixed, the results are qualitatively the same for different values of *w* (majority needed for anticonformity), i.e. functions *p*(*m*) have the same shape, although values are not identical, i.e. there are quantitative differences between them. This means the parameter *r* influences the results more strongly than the parameter *w*.

**Fig 4:**
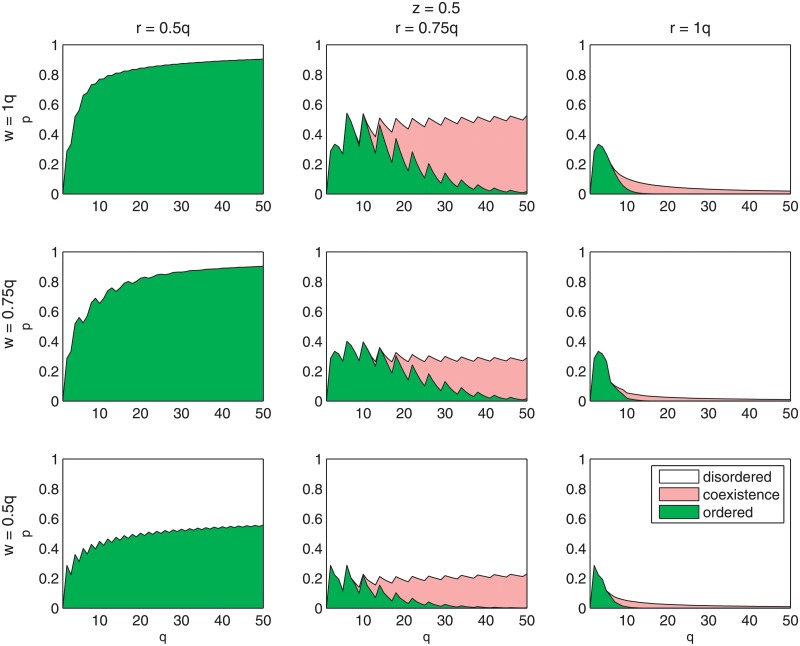
Phase diagram for the threshold *q*-voter model with two types of nonconformity in a (*p*, *q*) space. For the conformity threshold *r* = 0.5*q* (first column) only continuous order-disorder phase transitions are possible, whereas for *r* ≥ 0.75*q* the type of the phase transition depends on *q*. For small values of *q* again only continuous order-disorder phase transitions are possible, whereas for *q* > q* discontinuous phase transitions appear and there is an area in which a disordered phase coexists with the ordered one.

This result can be also discussed in context of Fig 4. On Fig 4 phase diagrams are presented for the same values of *r*, *w* and *z* as in Fig 3. Again, we see that all subplots within each column are qualitatively similar to each other, which means that as long as the value of *r* (the majority needed for conformity) is fixed, the results are qualitatively the same. Black lines in this figure represent the dependencies between the critical level of nonconformity *p* = p* and the size of the group of influence *q*. It is seen that only in the first column of Fig 4, i.e. for *r* = 0.5*q*, there is a single line in each subplot, which indicates continuous phase transition. Above these lines, i.e., for values of *q* and *p* which correspond to white areas of subplots, there is a stalemate state with public opinion *m* = 0 and below the critical line p* = p*(*q*) (green region) average opinion *m* ≠ 0.

In the second (*r* = 0.75*q*) and the third column (*r* = *q*), there are two transition lines and three different regions, which indicates a discontinuous phase transition. The saw-like behavior, seen particularly clear for *r* = 0.75*q*, is a trivial effect caused by the selection of *r* and *w* values as a fractions of *q* and necessity for them to be an integer. So, in fact there is a hidden continuous parameter *a*, being a real fraction of *q* and parameters *r* and *w* take integer values ⌈*a* ⋅ *q*⌉.

The lines, so-called spinodals, are limits of stability of a given phase. Above the upper spinodal line (white region), only the stalemate situation is stable, and thus the system will eventually reach the steady state with *m* = 0. Below the lower spinodal line (green region), the system will evolve towards the steady state with *m* ≠ 0 (majority of one opinion). The most intriguing region is that between the spinodal lines because it corresponds to metastability, and the final state is determined by the initial condition. In the finite-size systems, such as social systems, the situation is even more interesting because the system can spontaneously change from the ordered to the disordered state. Both phenomena, phase coexistence and the dependence on the initial conditions (so called hysteresis) are signatures of discontinuous phase transitions. With decreasing *q* spinodal lines are approaching each other and finally for *q* = q* (so called tricritical point) they collapse into a single line of continuous phase transition.

So far, we have discussed results only for *z* = 0.5. We expect that this parameter will also strongly influence the type of phase transition, because it allows us to tune the model from Model I (conformity + independence), for which both types of transitions are observed, to Model A (conformity + anticonformity) with only continuous phase transitions regardless of *q*. As a result of this tuning a critical value of *z* = z* appears, for *z* < z* transitions are continuous, whereas for *z* > z* there are discontinuous. As expected z* depends on all model’s parameters but unexpectedly this dependence is very complex and particularly interesting from the social point of view.

**Fig 5:**
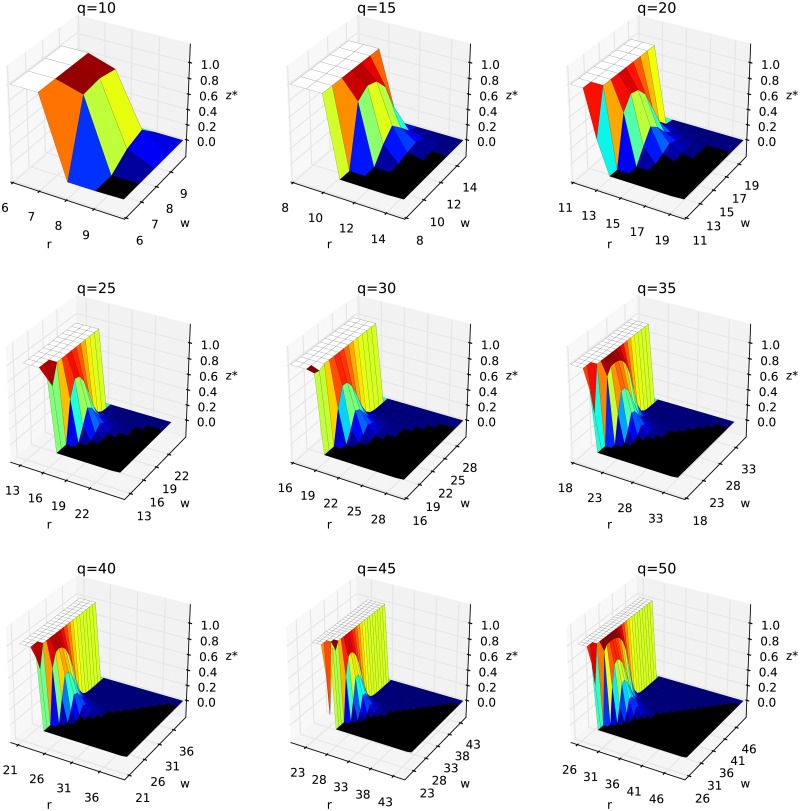
Critical value of the independence threshold *z* = z* for different values of *q*. For *z* < z* the phase transition is continuous, whereas for *z* > z*, it is discontinuous. The white color corresponds to the region where transition is always continuous (z* = 1), whereas the black color stands for discontinuity (z* = 0). In colored region, the transition can be either discontinuous or continuous, depending on *z*.

It is seen in Fig 5 that there is a critical value of majority for conformity *r* = r* below which the transition is always continuous regardless of the value of *w* and *z*, which means z* = 1. This critical value r* increases with decreasing *q*, which is another interesting result from the social point of view. For example for *q* = 10 (first column, first raw in Fig 5) r* = 8, which gives the value of critical majority r*/*q* equal to 80%, for *q* = 15 (second column, first raw in Fig 5) r* = 11, i.e. critical majority equal to 0.73%, and for the largest *q* presented here, i.e. *q* = 50 the threshold r*/*q* = 0.6. It is seen that r*/*q* decreases with *q*, and for the infinite *q* it will reach unfortunately the trivial value 0.5. The question, for which we do not have an answer, whether *q* → ∞ is realistic from a social point of view with the reference to the descriptive norms?

In Fig 5 it is also seen again that parameter *w*, describing the majority for anticonformity, has almost no influence on the results, which will be further discussed below from a social-psychological point of view. This non-effect is seen particularly clearly if one looks at the white areas in Fig 5, which denote regions with only continuous phase transitions regardless of *z*, i.e. where z* = 1.

## Discussion

In the Introduction, we asked whether there is a critical value of majority needed for conformity. We have not defined in what sense it should be critical, but only referenced several social experiments in which different values of a majority had been used. Asch has shown that only a unanimous majority is reliably powerful in yielding conformity [6], and indeed, many later lab experiments on conformity used only unanimous majorities [7]. However, in all these experiments rather small groups, which corresponds in our model to small value of *q*, were used. In case of descriptive norms, as was used in the environmental studies on energy consumptions or reusing towels [1, 2], the target is influenced by a virtual but potentially large group of people and thus a unanimous majority would be unrealistic. Thus, the question is if there is any critical value related to the threshold needed for social influence.

In our model, we have examined two such thresholds: (1) OTR for conformity, denoted by *r*, describing the ratio of people in a source of influence needed for conformity and (2) OTR for anticonformity, denoted by *w*, describing the ratio of people in a source of influence needed for aniconformity. We have proposed a possibly general model so *r* ≠ *w* and both parameters could take arbitrary values. Although we introduced conformity and anticonformity to the model identically on a conceptual level, which agrees with the social–psychological literature [13, 17], we found, surprisingly, that *r* influences the results significantly more than *w*. Perhaps most importantly, a critical value of *r* does indeed exist. Moreover, we found that the critical value of *r* decreases with the increasing size of the source of influence *q*, which is also consistent with social–psychological research. Summarizing, we have obtained within the model that:

1. The threshold *r*, related to conformity has a significant impact on the opinion dynamics. If the Other-Total Ratio *r* is below a certain critical value, then the order-disorder phase transition driven by the probability of nonconformity *p* is continuous. This means that the transition—between the system with one dominating opinion and the completely polarized system (i.e. both opinions have the same amount of support)—is continuous and smooth. With increasing nonconformity *p*, domination of one opinion over the other gradually decreases to zero. On the other hand, if OTR for conformity is above a certain critical value, the transition becomes discontinuous. This means that the transition between the system with one dominating opinion to the completely polarized system is abrupt. For a certain level of nonconformity, there is a jump from relatively large dominance of one opinion over the other to zero. Moreover, the value at which the jump occurs and the size of the jump depend on the initial conditions, which is exclusive for discontinuous phase transitions.
2. The critical OTR for conformity depends on the size of the group of influence, specifically, it decreases with the size of the group *q*, which seems to be a socially reliable result. For example r*(*q* = 10) = 0.8 and r*(*q* = 15) = 0.73.
3. The Other-Total ratio *w*, related to anticonformity, influences the results much more weakly than the OTR for conformity *r*.

The above results are also very interesting from the agent-based modeling point of view. The common critique of these kinds of models is that “what you put in is what you get.” This critique may seem paradoxical, however, because the essence of complex systems is emergence, which means that the system, which consists of many interacting elements (e.g. people, in case of social systems), exhibits qualitatively new behavior that is not a simple sum of individuals’ behaviors. One of the most spectacular types of an emergent behavior is the phase transition, which is present in this model. Such a transition is not unexpected, however, because lots of past research has taught us that we can expect phase transitions whenever a disordering force (here nonconformity) competes with an ordering force (here conformity). What cannot be easily predicted is the type of phase transition. Hence, the existence of the critical threshold for conformity *r* = r*(*q*) is the first macro surprise that emerges from our microscopic model. Even more striking in our opinion, however, is the fact that only the threshold for conformity *r* significantly influences the macroscopic outcome of the model, whereas the impact of the threshold *w* for anticonformity is nearly nonexistent. Is this result reasonable from the perspective of social psychology? Although most social psychologists have claimed that anticonformity and conformity are just two faces of the same coin, at the operational level, that is, opposites with the respect to the influence target’s postinfluence direction of movement. With conformity, the target moves towards the influence source, but with anticonformity against the source. At the conceptual level, however, the responses are similar in that both indicate behavior that is dependent upon the source or group, that is, behavior that has been affected by the source [17]. In our model, this is also true. So why is that the threshold for conformity should be so much more important than that for anticonformity?

First, let us try to answer this question from a social–psychological point of view. It is true that in the case of both conformity and anticonformity targets of influence pay attention to others. However, the motives for these two behaviors are very different, and this could be the reason why a threshold exists for conformity but not for anticonformity. To the best of our knowledge, there are only five well-defined and supported reasons for conformity: (a) to be correct, (b) to be socially accepted, (c) to accomplish group goals, (d) to establish or maintain one’s self-concept or social identity, and/or (e) to align one’s self with similar or liked others or with fellow in-group members—in this vein, it is psychologically consistent to agree with others towards whom one feels similar, likes, or feels a part [16]. Note: each of these motives is related to the reference group, the source of influence; hence, one might very well expect the existence of a threshold for conformity in these types of circumstances. Motives for anticonformity, however, are much more diverse and not necessarily related to a primary reference group. These motives include the desire (a) to provoke intra-group disharmony, as in the case of trying to force a change in leadership or destroy a group, (b) to separate one’s self from one or more dissimilar, disliked, or unattractive others or from out-group members, (c) to establish or project one’s behavioral freedom/autonomy, (d) to establish or project one’s uniqueness, (e) to avoid “Groupthink”—rapid foreclosure in group decision making, that is, without thoughtful, deliberate processes and debate, often in support of a powerful or charismatic leader, and/or (f) to avoid the appearance of sycophancy [16]. This list indicates that only some motives for anticonformity are related to the group of influence, while others are not. What is more, it could be that it is the interplay between all of these factors that results in the lack of a threshold with respect to anticonformity.

However, this difference between conformity and anticonformity, that is based on different motives for these types of response, does not exist in our model. Therefore, although our results have been obtained analytically and seem reasonable from a social-psychological point of view, it is not easy to explain them heuristically within the present model. This is what we call the *macro-surprise despite complete micro-level knowledge* [53].

## Supporting information

S1 Appendix Derivation of an effective potential and Landau’s approach. (PDF)
