# Supplementary material for: Conformity in numbers—Does criticality in social responses exist?
Source: PLoS One. 2018 Dec 27;13(12):e0209620. doi: 10.1371/journal.pone.0209620 (PMC6307709; doi:10.1371/journal.pone.0209620)
Supplement: S1 Appendix — (PDF) [file pone.0209620.s001.pdf]

Conformity in numbers – does criticality in social responses exist?

Piotr Nyczka, Katarzyna Byrka, Paul R. Nail, Katarzyna Sznajd-Weron

Supporting information: S1 Appendix.

Derivation of an effective potential and Landau's approach.

The goal is to determine the critical point p^*, below the system is ordered and the tricritical point z^*, i.e. such a value of probability of independence that for $z < z^*$ transition is continuous, but for $z > z^*$ discontinuous. We will use Landau's approach and therefore first we need to derive an effective potential in term of the order parameter m. To do this we first need to rewrite the conditional probabilities of conformity $\alpha^{\pm}$, anticonformity $\beta^{\pm}$ and independence $\gamma^{\pm}$ in term of the order parameter m:

$$\begin{aligned}
\alpha^{+} &= \frac{1}{2}^{q+1} \sum_{i=r}^{q} \sum_{k=0}^{i} \sum_{l=0}^{q-i+1} \binom{q}{i} \binom{i}{k} \binom{q-i+1}{l} (-1)^{l} m^{k+l}, \qquad (1) \\
\alpha^{-} &= \frac{1}{2}^{q+1} \sum_{i=r}^{q} \sum_{k=0}^{i} \sum_{l=0}^{q-i+1} \binom{q}{i} \binom{i}{k} \binom{q-i+1}{l} (-1)^{k} m^{k+l}, \\
\beta^{+} &= \frac{1}{2}^{q+1} \sum_{i=r}^{q} \sum_{k=0}^{i+1} \sum_{l=0}^{q-i} \binom{q}{i} \binom{i+1}{k} \binom{q-i}{l} (-1)^{k} m^{k+l}, \\
\beta^{-} &= \frac{1}{2}^{q+1} \sum_{i=r}^{q} \sum_{k=0}^{i+1} \sum_{l=0}^{q-i} \binom{q}{i} \binom{i+1}{k} \binom{q-i}{l} (-1)^{l} m^{k+l}, \\
\gamma^{+} &= \frac{1-m}{4}, \\
\gamma^{-} &= \frac{1+m}{4}.
\end{aligned}$$

Furthermore we define:

$$\begin{aligned}
\alpha &\equiv \alpha^{+} - \alpha^{-}, \\
\beta &\equiv \beta^{+} - \beta^{-}, \\
\gamma &\equiv \gamma^{+} - \gamma^{-}. \qquad (2)
\end{aligned}$$

$$(3)$$

By combining (2) and (3) we obtain:

$$\begin{aligned}
\alpha &= \frac{1}{2}^{q+1} \sum_{i=r}^{q} \sum_{k=0}^{i} \sum_{l=0}^{q-i+1} \binom{q}{i} \binom{i}{k} \binom{q-i+1}{l} ((-1)^{l} - (-1)^{k}) m^{k+l}, \qquad (4) \\
\beta &= \frac{1}{2}^{q+1} \sum_{i=r}^{q} \sum_{k=0}^{i+1} \sum_{l=0}^{q-i} \binom{q}{i} \binom{i+1}{k} \binom{q-i}{l} ((-1)^{k} - (-1)^{l}) m^{k+l}, \\
\gamma &= -\frac{m}{2}.
\end{aligned}$$

Above formulas contain the following powers of the order parameter m^{k+l}, and therefore for a given $m^{(k+l)}$ we can claim $(k+l)$ to be constant. Let's then rewrite expression (5) with respect to the order of m. Due to the antisymmetry of α, β and γ, odd terms vanish. Therefore if we put $2n-1 = k+l$, where $n = 1, 2, 3, ..., \lfloor q/2 \rfloor + 1$ we can write:

$$\begin{aligned} \alpha &= -\frac{1}{2}^q \sum_{n=1}^{\lfloor q/2 \rfloor+1} \sum_{i=r}^{q} \sum_{k=0}^{2n-1} \binom{q}{i} \binom{i}{k} \binom{q-i+1}{2n-1-k} (-1)^k m^{2n-1}, \\ \beta &= \frac{1}{2}^q \sum_{n=1}^{\lfloor q/2 \rfloor+1} \sum_{i=r}^{q} \sum_{k=0}^{2n-1} \binom{q}{i} \binom{i+1}{k} \binom{q-i}{2n-1-k} (-1)^k m^{2n-1}, \\ \gamma &= -\frac{m}{2}, \end{aligned} \tag{5}$$

and terms of particular order will be:

$$\begin{aligned} \alpha_n &= -\frac{1}{2}^q \sum_{i=r}^{q} \sum_{k=0}^{2n-1} \binom{q}{i} \binom{i}{k} \binom{q-i+1}{2n-1-k} (-1)^k m^{2n-1}, \\ \beta_n &= \frac{1}{2}^q \sum_{i=r}^{q} \sum_{k=0}^{2n-1} \binom{q}{i} \binom{i+1}{k} \binom{q-i}{2n-1-k} (-1)^k m^{2n-1}, \\ \gamma_n &= \begin{cases} -\frac{m}{2} & \text{for} \quad n = 1, \\ 0 & \text{for} \quad n > 1, \end{cases} \end{aligned} \tag{6}$$

where the number of term is n and order of term is $2n-1$. Now we can define force F and potential V as:

$$\begin{aligned} F(m) &= \lambda^+ - \lambda^- = (1-p)\alpha + p(1-z)\beta + pz\gamma, \\ V(m) &= -\int F(m) dm. \end{aligned} \tag{7}$$

As usually in the Landau's approach we use Taylor's expansion around $m = 0$. Because potential V is symmetric, only even terms survive:

$$F \approx A(p,z)m + B(p,z)m^3 + C(p,z)m^5, \tag{8}$$

$$V \approx -\frac{1}{2}A(p,z)m^2 - \frac{1}{4}B(p,z)m^4 - \frac{1}{6}C(p,z)m^6, \tag{9}$$

where:

$$\begin{aligned} A(p,z)m &= (1-p)\alpha_1 + p(1-z)\beta_1 + pz\gamma_1, \\ B(p,z)m^3 &= (1-p)\alpha_2 + p(1-z)\beta_2 + pz\gamma_2, \\ C(p,z)m^5 &= (1-p)\alpha_3 + p(1-z)\beta_3 + pz\gamma_3. \end{aligned} \tag{10}$$

To determine type of the phase transition we need to look at the potential for critical $p = p^*$. In this point $\frac{\partial^2 V(p^*,z^*)}{\partial m^2} = 0$ for $m = 0$ therefore A vanishes. Then we have to look for such a $z = z^*$ where $\frac{\partial^4 V(p^*,z^*)}{\partial m^4} = 0$ for $m = 0$ it means that we need B to vanish also:

$$\begin{aligned} A(p^*, z^*) &= 0, \\ B(p^*, z^*) &= 0. \end{aligned} \tag{11}$$

From the above condition we derive:

$$p^* = \frac{\alpha_1\beta_2 - \alpha_2\beta_1 + \alpha_2\gamma_1}{\alpha_1\beta_2 - \alpha_2\beta_1 + \alpha_2\gamma_1 - \beta_2\gamma_1}, \tag{12}$$

$$z^* = \frac{\alpha_1\beta_2 - \alpha_2\beta_1}{\alpha_1\beta_2 - \alpha_2\beta_1 + \alpha_2\gamma_1}.$$

We can expand (6) as:

$$\begin{aligned}
\alpha_1 &= -\frac{1}{2}^q \sum_{i=r}^{q} \binom{q}{i} \left[\binom{i}{0}\binom{q-i+1}{1} - \binom{i}{1}\binom{q-i+1}{0} \right], \qquad (13)\\
\alpha_2 &= -\frac{1}{2}^q \sum_{i=r}^{q-2} \binom{q}{i}\binom{i}{0}\binom{q-i+1}{3},\\
&\quad +\frac{1}{2}^q \sum_{i=r}^{q-1} \binom{q}{i}\binom{i}{1}\binom{q-i+1}{2},\\
&\quad -\frac{1}{2}^q \sum_{i=r}^{q} \binom{q}{i} \left[\binom{i}{2}\binom{q-i+1}{1} - \binom{i}{3}\binom{q-i+1}{0} \right],\\
\beta_1 &= +\frac{1}{2}^q \sum_{i=r}^{q-1} \binom{q}{i}\binom{i+1}{0}\binom{q-i}{1},\\
&\quad -\frac{1}{2}^q \sum_{i=r}^{q} \binom{q}{i}\binom{i+1}{1}\binom{q-i}{0},\\
\beta_2 &= \frac{1}{2}^q \sum_{i=w}^{q-3} \binom{q}{i}\binom{i+1}{0}\binom{q-i}{3},\\
&\quad -\frac{1}{2}^q \sum_{i=w}^{q-2} \binom{q}{i}\binom{i+1}{1}\binom{q-i}{2},\\
&\quad +\frac{1}{2}^q \sum_{i=w}^{q-1} \binom{q}{i}\binom{i+1}{2}\binom{q-i}{1},\\
&\quad -\frac{1}{2}^q \sum_{i=w}^{q} \binom{q}{i}\binom{i+1}{3}\binom{q-i}{0},\\
\gamma_1 &= -\frac{1}{2},\\
\gamma_2 &= 0,
\end{aligned}$$

and then after simple algebraic transformations we get final formulas:

$$\begin{aligned}
\alpha_1 &= -\left(\frac{1}{2}\right)^q \sum_{i=r}^{q} \binom{q}{i}(q-2i+1), \qquad (14)\\
\alpha_2 &= \frac{1}{6}\left(\frac{1}{2}\right)^q \binom{q}{r} r(q^2-4qr+4q+4r^2-10r+5),\\
\beta_1 &= -\left(\frac{1}{2}\right)^q \left[q+1+\sum_{i=w}^{q-1}\binom{q}{i}(2i-q+1) \right],\\
\beta_2 &= \frac{1}{6}\left(\frac{1}{2}\right)^q \binom{q}{w} w(-q^2+4qw+2q-4w^2-2w+1),\\
\gamma_1 &= -\frac{1}{2},\\
\gamma_2 &= 0.
\end{aligned}$$

Having above formulas we were able to determine tricritical point $z^* = z^*(q)$ with precision up to the order 10^{-4}, depending on the q. The higher q the greater precision.
